# Inhibition of phosphodiesterases 1 and 4 prevents myofibroblast transformation in Peyronie's disease

**DOI:** 10.1111/bju.16631

**Published:** 2024-12-23

**Authors:** Sophie L. Harding, Marcus M. Ilg, Stephen A. Bustin, David J. Ralph, Selim Cellek

**Affiliations:** ^1^ Fibrosis Research Group, Medical Technology Research Centre Anglia Ruskin University Chelmsford UK; ^2^ Molecular Diagnostics Unit, Medical Technology Research Centre Anglia Ruskin University Chelmsford UK; ^3^ Urology Department University College London London UK

**Keywords:** Peyronie's disease, fibroblast, myofibroblast, tunica albuginea, fibrosis, phosphodiesterase

## Abstract

**Objectives:**

To investigate which phosphodiesterase (PDE) isoforms are expressed in fibroblasts isolated from the tunica albuginea (TA) of patients with Peyronie's disease (PD), and to measure the potency of PDE inhibitors in preventing transformation of these fibroblasts to profibrotic myofibroblasts.

**Materials and Methods:**

Fibroblasts isolated from the TA of men undergoing surgery for correction of PD curvature were transformed to myofibroblasts using transforming growth factor beta‐1. The expression of 21 PDE isoforms was investigated using quantitative reverse transcriptase‐polymerase chain reaction and protein analysis, as were the effects of various PDE inhibitors on prevention of myofibroblast transformation. Intracellular cAMP and cGMP in the presence of PDE inhibitors were quantified using cGMP/cAMP enzyme‐linked immunosorbant assay assays.

**Results:**

We found that PDE1A, 1C, 4, 5A, 7B and 8B were expressed at mRNA and protein levels. Selective inhibitors of these enzymes prevented myofibroblast transformation in a concentration‐dependent manner, with PDE1 inhibitor ITI‐214 and PDE4 inhibitors roflumilast and roflumilast N‐oxide showing greatest potency. ITI‐214 and roflumilast N‐oxide increased intracellular cAMP, but not cGMP, in a concentration‐dependent manner.

**Conclusions:**

This is the first demonstration of the expression of PDE1, 7 and 8 isoforms, and the function of PDE1 and PDE4 in human TA fibroblasts. The ability of inhibitors of these enzymes to prevent myofibroblast transformation suggests that such inhibitors can be developed to treat acute PD.

**Abbreviations**
IC_50_
: half‐maximal inhibitory concentration
PD: Peyronie's disease
PDE: phosphodiesterase
SMA: smooth muscle actin
TA: tunica albuginea
TGF‐β1: transforming growth factor beta‐1

## Introduction

Peyronie's disease (PD) is a fibrotic disorder of penile tunica albuginea (TA) that can lead to penile curvature, shortening and erectile dysfunction, with prevalence ranging from 0.38% to 22.5% depending on underlying risk factors such as age and diabetes [1]. Typically, patients with PD present in the acute (early) phase, usually within 6 months of the first symptoms such as pain during erection with or without a palpable penile mass. During this phase, it is thought that repetitive microtrauma triggers inflammation, which activates the fibrotic process. In this acute phase, there is no efficacious treatment available to patients. The chronic (late) phase of PD is characterised by the continuing excessive production of extracellular matrix proteins and tissue contraction, and subsequent calcification of the fibrotic plaque, which leads to curvature. In this phase, patients can only be offered collagenase injection and/or surgery [2]. There is therefore an urgent unmet need to develop novel therapeutics that can halt the progression of fibrosis in the early phase and that can reverse fibrosis in the late phase.

The fibrotic process in PD is centred around the transformation of resident fibroblasts to profibrotic myofibroblasts, which have increased proliferation, enhanced capacity, ability to produce large amounts of extracellular matrix proteins, and a gained ability to contract. The extracellular matrix proteins, produced in excessive amounts and deposited in a disorganised fashion by the myofibroblasts, are then remodelled into a dense fibrotic plaque that causes the penile curvature [3]. The prevention of transformation of resident fibroblasts to profibrotic myofibroblasts has been suggested as a therapeutic approach to prevent or slow progress of fibrosis [4]. One of the molecular pathways that would be an attractive target for drug discovery to inhibit myofibroblast transformation is the phosphodiesterase (PDE) pathway.

Phosphodiesterases are members of a highly conserved superfamily of enzymes, with 21 different genes and more than 50 mRNAs grouped into 11 broad families (PDE1–PDE11) that degrade the intracellular messenger cyclic nucleotides cAMP and/or cGMP. cAMP and cGMP are involved in the regulation of several physiological responses, such as smooth muscle contraction, apoptosis, lipid metabolism and inflammation. PDE inhibitors increase the intracellular concentrations of cAMP/cGMP by inhibiting their metabolism. Several PDE inhibitors are used clinically for the treatment of various diseases: PDE3 inhibitors are used to prevent postoperative thrombosis, and for the treatment of heart failure; PDE4 inhibitors are used for the treatment of psoriasis, psoriatic arthritis and atopic dermatitis; and PDE5 inhibitors are used for erectile dysfunction and pulmonary arterial hypertension [5].

In the fibrosis field, there has been some research supporting the use of PDE inhibitors in the prevention and/or treatment of fibrosis, although none have reached the clinic. PDE1 inhibition has been suggested to exert antifibrotic effects in the heart [6]. PDE4 inhibitors showed anti‐inflammatory and antifibrotic effects in the lung [7] and skin [8]. A PDE4B inhibitor prevented lung function decrease in patients with idiopathic pulmonary fibrosis [9]. PDE5 inhibitors have been suggested to be antifibrotic in the heart [10] and lung [11].

In the PD field, whilst PDE4 and PDE5 have been shown to be expressed in human TA fibroblasts and myofibroblasts [12, 13], to date, no published studies have demonstrated antifibrotic activity of selective PDE4 inhibitors in PD fibroblasts. PDE5 inhibitors have been shown to have some antifibrotic effect in animal models of PD [13, 14] as well as in clinical studies [15, 16, 17]. So far, PDE5 inhibitors have not been licensed for the treatment of PD.

Currently, it is not known which PDEs other than PDE4 and 5 are expressed in human TA fibroblasts and whether inhibition of the PDEs that are expressed could prevent transformation of human TA‐derived fibroblasts to myofibroblasts. A better understanding of the expression levels of these enzymes and their function and pharmacology in target cells isolated from disease‐relevant tissue would support the development of novel PDE inhibitors for the treatment of PD.

## Materials and Methods

### Sample Acquisition and Fibroblast Isolation

Non‐fibrotic TA tissue samples were obtained from patients undergoing corrective surgery for PD curvature (the Nesbit procedure) at University College London Hospital. All tissue specimens were excised from the opposite side to the PD plaque, tissue that would otherwise be discarded. The tissue samples used in this study were obtained from seven patients, aged 33–62 years, with comorbidities of circumcision, hypertension, hyperlipidaemia, right inguinal hernia, diabetes, sleep apnoea, and smoking, and co‐medications including citalopram, statins, ramipril and metformin. The project has obtained approval from the NHS Research Ethics Committees (East of England 12/EE/0170 and North of Scotland 15/NS/0051), the Health Research Authority and the Anglia Ruskin University Faculty Research Ethics Panel (15/044), with all participating patients providing fully informed written consent to take part in the study.

Tissue samples were carefully dissected to ensure that only TA tissue remained. Fibroblasts were isolated using the explant technique, as described previously [13, 18]. Briefly, TA tissue samples were anchored into six‐well tissue culture plates and submerged in DMEM/F‐12 medium with 10% foetal calf serum and 1% penicillin–streptomycin. Samples were incubated at 37°C, 5% CO2 in a humidified atmosphere until cell outgrowth was observed. Tissue samples were removed, and passages 2–6 of the cells were used for experiments. The cells were confirmed as fibroblasts using immunocytochemistry as they were α‐smooth muscle actin (SMA)‐negative, vimentin‐positive and desmin‐negative, as previously shown [13, 18].

### Real‐Time Reverse Transcription‐Quantitative PCR

Cells were seeded into six‐well plates at 50 000 cells/well and left to attach overnight. Cells were left untreated or treated with 10 ng/mL transforming growth factor beta‐1 (TGF‐β1) for 72 h. Total RNA was extracted using RNeasy Mini Kits (Qiagen, UK). A DNAse digestion step was included to eliminate genomic DNA contamination (RNAse‐free DNAse set; Qiagen, UK). RNA quality was assessed using the Agilent 2100 Bioanalyser. All RNA samples had an RNA integrity number of 9 or above. RNA samples were converted to cDNA using the UltraScript 2.0 reverse transcriptase kit (PCR Biosystems, UK). Real‐time reverse transcriptase‐quantitative PCR (RT‐qPCR) was carried out using the SensiFAST*™* SYBR PCR kit (Bioline, London, UK) using the following protocol: polymerase activation at 95°C for 1 min, followed by 40 cycles at 95°C for 1 s and then 60°C for 1 s. Melt curves were generated by holding at 95°C for 1 s, 65°C for 1 s, gradually increasing to 95°C, and holding at 95°C for 1 s. RNA samples were run in triplicate, with a no template cDNA negative control. *GAPDH* was used as the reference gene as its expression was shown not to be affected by any of the treatments. The primer list is shown in Table S1.

### Gene Expression Likelihood Assessment

For each gene tested, mean Cq values were calculated, and melt curve quality was assessed. With these data, the flow chart in Fig. S1 was used to determine the likelihood of expression at biologically relevant levels per gene, the outcomes being one of three: ‘likely’, ‘possibly’ or ‘unlikely’. Melt curves were determined to be of ‘good’ quality if a single, strong peak was present. A ‘poor’ melt curve, characterised by numerous small peaks across the temperature gradient, was indicative of that gene being unlikely to be expressed. For genes with a ‘good’ melt curve the mean Cqs for untreated and TGF‐β1‐treated cells were assessed; a Cq of below 30 for either condition was indicative of a gene that was likely to be expressed. Genes with Cqs above 30, but below 33 were determined to be possibly expressed. Genes with Cqs above 33 were deemed unlikely to be expressed at biologically relevant levels. Genes categorised as ‘likely’ or ‘possibly’ were taken forward for protein analysis.

### In‐Cell ELISA

Protein expression of α‐SMA and PDE isoforms were quantified as previously described [13, 18]. Cells were seeded into 96‐well microplates at 5000 cells/well and left to attach overnight. Cells were incubated with or without 10 ng/mL TGF‐β1, with or without PDE inhibitors, for 72 h. Cells were fixed with 4% paraformaldehyde and blocked with 10% donkey serum in PBS containing 0.1% Triton X‐100. Cells were incubated with primary antibodies (Table S2) for 2 h. Cells were washed, then incubated for 1 h with the nuclear stain DRAQ5 (1:000; Biostatus, UK) and a secondary antibody (Table S2). Plates were scanned using an infrared imaging system (Odyssey CLx imager; LI‐COR).

### cGMP and cAMP Assays

Cells were seeded into six‐well plates at 150 000 cells/well and incubated overnight. Media were replaced with fresh media or media containing 10 ng/mL TGF‐β1, ± compounds, and incubated for 10 min. Cells were washed with ice cold PBS and lysed with 250 μL ice cold 0.1 M HCl over 20 min. Cells were scrapped from the wells using cell scrappers. Cell suspension was vortexed for 10 s and centrifuged for 10 min at 14 000 *g* at 4°C. The supernatant was split into subsamples for the cGMP/cAMP assays and the protein quantification.

cGMP and cAMP concentrations were measured using ELISA kits (Abcam, UK). All samples were acetylated for the cGMP assay. Each sample was tested in triplicate. The plates were read on a CLARIOstar Plus microplate reader (BMG LABTECH, UK). A BCA Protein Assay kit (Bio‐Rad) was used for quantification of protein levels in each sample.

### Statistical Analysis

Data were analysed using Microsoft Excel 2013 and GraphPad Prism 10 software and presented as mean ± SEM. A Kolmogorov–Smirnov test revealed that the data were distributed normally. Student's *t*‐test for unpaired means (two‐sided) was used to compare two groups. Dunnett's multiple comparisons test was used to compare more than two groups. For both statistical tests, a *P* value less than 0.05 was taken to indicate statistical significance. Experiments were performed using samples from three patients (*N* = 3). At least three technical replicates were performed for each sample (*n* = 9).

## Results

### Likelihood of Expression of PDE Genes in the Presence and Absence of TGF‐β1

We used RT‐qPCR to investigate which PDE genes were expressed in TA‐derived fibroblasts with or without TGF‐β1 treatment. Genes determined as ‘likely’ to be expressed were *PDE1C*, *PDE3A*, *PDE4A*, *PDE4B*, *PDE5A*, *PDE7A*, *PDE7B* and *PDE8A*, and ‘possibly’ expressed genes were *PDE1A*, *PDE3B and PDE8B*. Genes ‘unlikely’ to be expressed were *PDE1B*, *PDE2A*, *PDE4C*, *PDE4D*, *PDE6A*, *PDE6B*, *PDE6C*, *PDE9A*, *PDE10A* and *PDE11A* (Table 1).

**Table 1:** Summary of likelihood of expression of phospodiesterases in tunica albuginea‐derived fibroblasts.

Gene	Mean Cq values (untreated cells)	Mean Cq values (TGF‐β1‐treated cells)	Melt curve quality	Likelihood of expression
*GAPDH*	19.6	20.7	Good	Likely
*PDE1A*	30.9	34.2	Good	Possibly
*PDE1B*	37.6	35.6	Poor	Unlikely
*PDE1C*	28.9	30.0	Good	Likely
*PDE2A*	34.8	35.0	Poor	Unlikely
*PDE3A*	29.5	32.1	Good	Likely
*PDE3B*	31.5	34.3	Good	Possibly
*PDE4A*	25.8	26.8	Good	Likely
*PDE4B*	26.9	28.8	Good	Likely
*PDE4C*	33.7	33.4	Poor	Unlikely
*PDE4D*	34.3	33.5	Good	Unlikely
*PDE5A*	27.2	31.8	Good	Likely
*PDE6A*	34.0	33.7	Poor	Unlikely
*PDE6B*	35.5	35.1	Poor	Unlikely
*PDE6C*	37.6	36.5	Poor	Unlikely
*PDE7A*	28.8	29.3	Good	Likely
*PDE7B*	27.7	28.6	Good	Likely
*PDE8A*	27.1	27.0	Good	Likely
*PDE8B*	31.7	32.2	Good	Possibly
*PDE9A*	34.6	34.8	Good	Unlikely
*PDE10A*	34.3	33.4	Good	Unlikely
*PDE11A*	36.5	35.5	Poor	Unlikely

Cells were exposed to 10 ng/mL TGF‐β1 or media only for 72 h before RNA isolation. RNA was quantified using reverse transcriptase‐quantitative PCR (RT‐qPCR). Cq values were derived from amplification plots generated during RT‐qPCR and can be defined as the PCR cycle at which the assay passed the fluorescence detection threshold. Melt curves were generated at the end of the RT‐qPCR run and determined to be of ‘good’ quality if a single, strong peak was present. Each gene was tested in three patient cell lines in triplicate. PDE, phosphodiesterase; TGF‐β1, transforming growth factor beta‐1.

### Expression of Selected PDE Proteins in the Presence and Absence of TGF‐β1

**Fig. 1:**
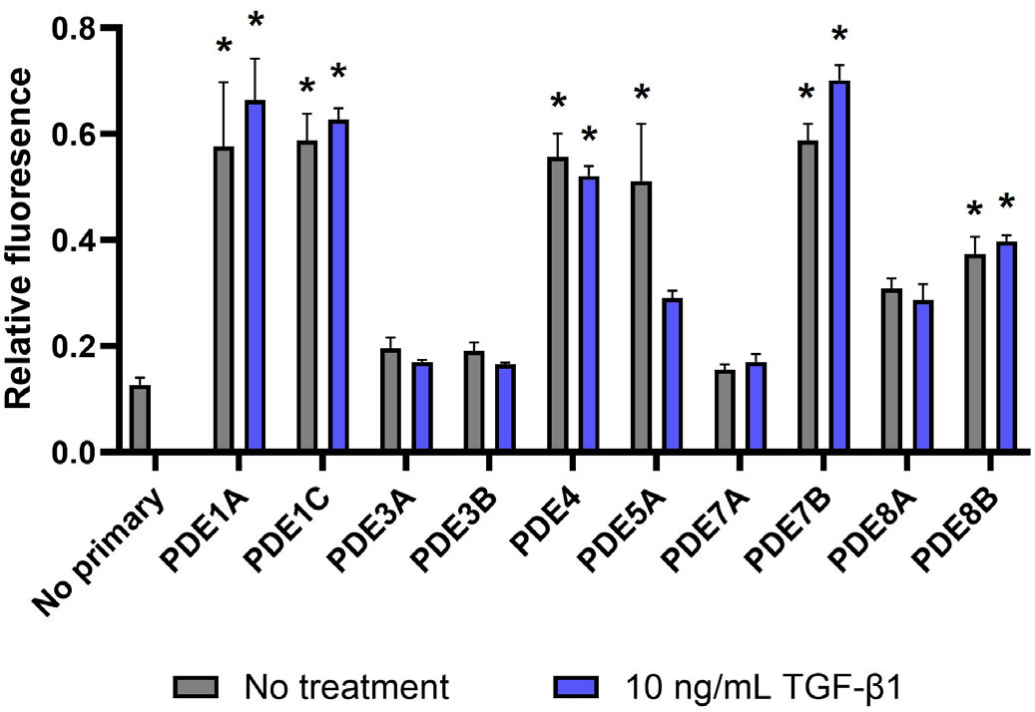
Protein expression of phosphodiesterase (PDE) proteins in tunica albuginea‐derived cells from patients with Peyronie's disease. Fibroblasts were exposed to transforming growth factor beta‐1 (TGF‐β1; 10 ng/mL) or blank media for 72 h and stained with secondary antibody raised against PDE1A, PDE1C, PDE3A, PDE3B, PDE4, PDE5A, PDE7A, PDE7B, PDE8A or PDE8B. Data were normalised as a ratio of relevant protein/DNA staining (700 nm fluorescence intensity / 800 nm fluorescence intensity). Data points are plotted as mean ± SEM, *N* = 3. *P < 0.05 significant statistical difference vs no primary antibody control using Dunnett's multiple comparisons test.

The PDE genes that were determined to be either ‘likely’ or ‘possibly’ expressed were taken forward for protein analysis. In the absence of TGF‐β1, PDE1A, PDE1C, PDE4, PDE5A, PDE7B and PDE8B were significantly (*P < 0.05) expressed compared to the control. In the presence of TGF‐β1, PDE1A, PDE1C, PDE4, PDE7B and PDE8B were significantly (*P < 0.05) expressed (Fig. 1).

### Selective Inhibition of PDE Enzymes

**Fig. 2:**
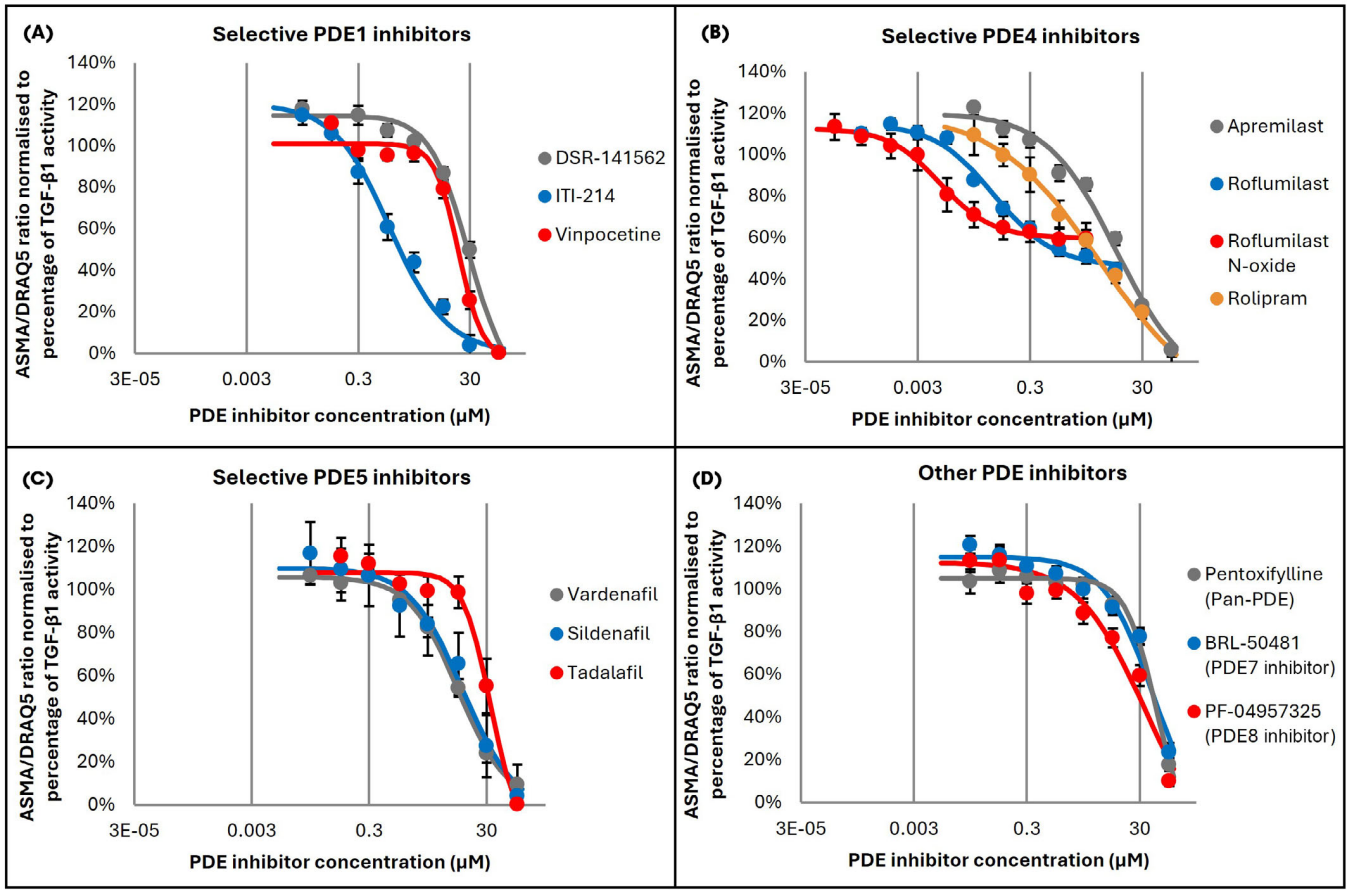
Effect of phosphodiesterase (PDE) inhibitors on transforming growth factor beta‐1 (TGF‐β1)‐induced myofibroblast transformation in tunica albuginea‐derived fibroblasts. **(A)** Cells were exposed to 10 ng/mL TGF‐β1 and a range of concentrations of PDE inhibitors for 72 h. Data were normalised as a ratio of α‐smooth muscle actin (SMA)/DNA staining (700 nm fluorescence intensity/800 nm fluorescence intensity) and presented as a percentage of TGF‐β1‐induced α‐SMA expression. **(A)** Concentration–response curves (CRCs) of selective PDE1 inhibitors DSR‐141562, ITI‐214 and vinpocetine. **(B)** CRCs of selective PDE4 inhibitors apremilast, roflumilast, roflumilast N‐oxide and rolipram. **(C)** CRCs of selective PDE5 inhibitors vardenafil, sildenafil and tadalafil. **(D)** CRCs of pan‐PDE inhibitor pentoxifylline, PDE7 inhibitor BRL‐50481, and PDE8 inhibitor PF‐04957325. Data points are plotted as mean ± SEM, *N* = 3, *n* = 9.

The data above suggest that PDE1, PDE4, PDE5, PDE7 and PDE8 isoforms are present in TA‐derived fibroblasts. To investigate which (if any) of these PDE isoforms play a role in myofibroblast transformation, concentration–response curves of selective PDE inhibitors were constructed to investigate their effect on TGF‐β1‐induced α‐SMA expression. All compounds tested inhibited TGF‐β1‐induced myofibroblast transformation in a concentration‐dependent manner (Fig. 2). Of the PDE1 inhibitors, ITI‐214 demonstrated the highest potency, with a half‐maximal inhibitory concentration (IC_50_) of 1.3 μM, while DSR‐141562 had an IC_50_ of 25.5 μM, and vinpocetine an IC_50_ of 18.9 μM (Fig. 2A). PDE4 inhibitors roflumilast and roflumilast N‐oxide showed high potency with IC_50_ values of 0.063 and 0.009 μM, respectively, and apremilast and rolipram had respective IC_50_ values of 9.7 and 5.1 μM (Fig. 2B). PDE5 inhibitors vardenafil, sildenafil and tadalafil inhibited myofibroblast transformation, with IC_50_ values of 10.2, 12.1 and 34.9 μM, respectively (Fig. 2C). The remaining PDE inhibitors demonstrated the lowest potency, with the pan‐PDE inhibitor pentoxifylline displaying an IC_50_ of 60.7 μM, the PDE7 inhibitor BRL‐50481 an IC_50_ of 47.6 μM, and the PDE8 inhibitor PF‐04957325 an IC_50_ of 34.8 μM (Fig. 2D).

### cGMP and cAMP Assays

**Fig. 3:**
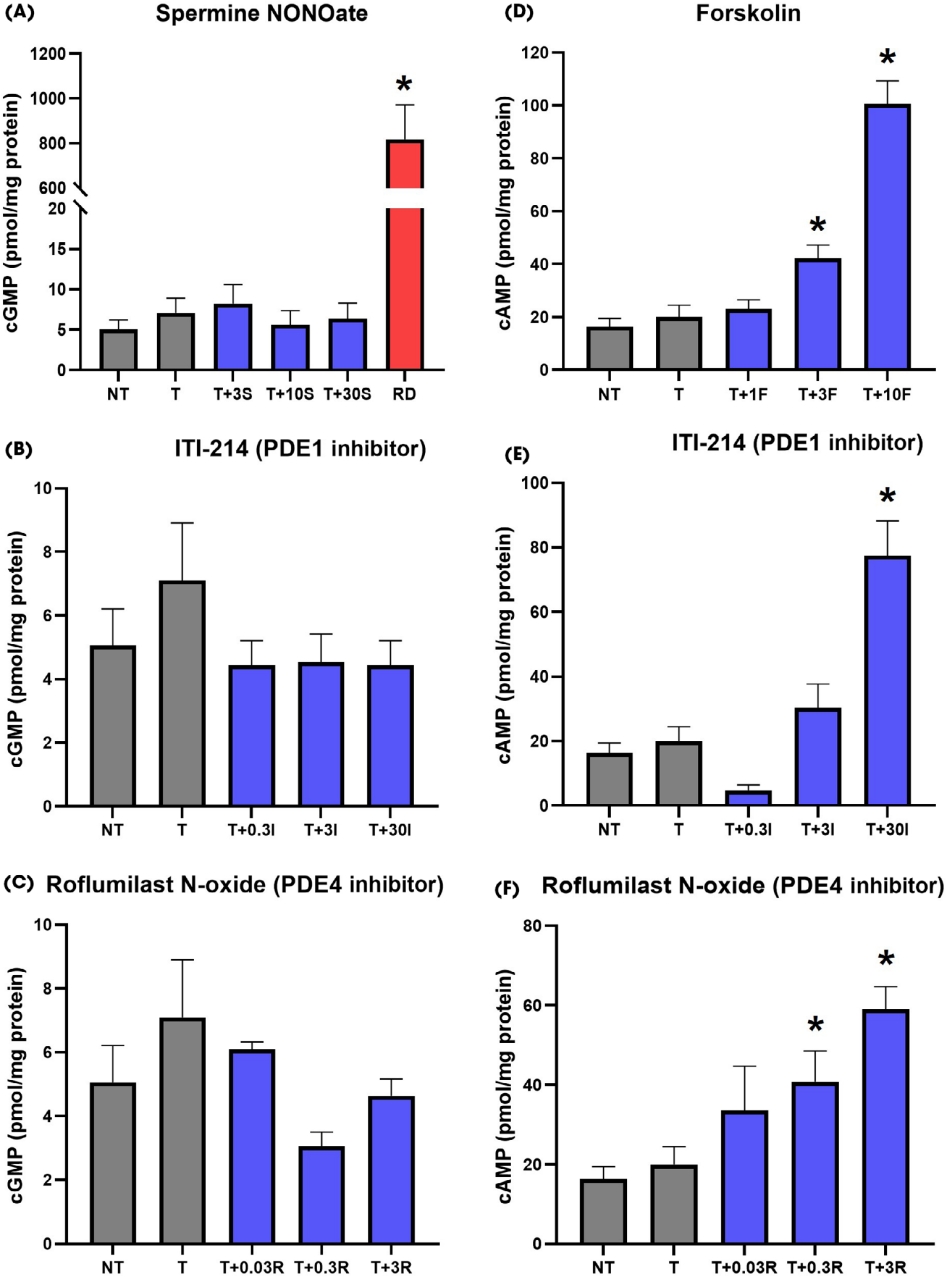
cGMP and cAMP detected from lysed tunica albuginea (TA)‐derived fibroblasts treated with transforming growth factor beta‐1 (TGF‐β1), plus spermine NONOate, forskolin, or phosphodiesterase (PDE) inhibitors. **(A)** cGMP levels in fibroblasts, which were treated with blank media (no treatment [NT]), 10 ng/mL TGF‐β1 (treatment [T]), 10 ng/mL TGF‐β1 plus nitric oxide donor 3 μM spermine NONOate (T + 3S), 10 μM spermine NONOate (T + 10S) or 30 μM spermine NONOate (T + 30S) and in rhabdomyosarcoma cells, which were treated with 10 μM spermine NONOate (rhabdomyosarcoma). **(B)** cGMP levels in fibroblasts were treated with blank media (NT), 10 ng/mL TGF‐β1 (T), 10 ng/mL TGF‐β1 plus 0.3 μM PDE1 inhibitor ITI‐214 (T + 0.3I), 3 μM ITI‐214 (T + 3I) or 30 μM ITI‐214 (T + 30I). **(C)** cGMP levels in fibroblasts were treated with blank media (NT), 10 ng/mL TGF‐β1 (T), 10 ng/mL TGF‐β1 plus 0.03 μM PDE4 inhibitor roflumilast N‐oxide (T + 0.03R), 0.3 μM roflumilast N‐oxide (T + 0.3R) or 3 μM roflumilast N‐oxide (T + 3R). **(D)** cAMP levels in fibroblasts were treated with blank media (NT), 10 ng/mL TGF‐β1 (T), 10 ng/mL TGF‐β1 plus 1 μM forskolin (T + 1F), 3 μM forskolin (T + 3F) or 10 μM forskolin (T + 10F). **(E)** cAMP levels in fibroblasts were treated with blank media (NT), 10 ng/mL TGF‐β1 (T), 10 ng/mL TGF‐β1 plus 0.3 μM PDE1 inhibitor ITI‐214 (T + 0.3I), 3 μM ITI‐214 (T + 3I) or 30 μM ITI‐214 (T + 30I). **(F)** cAMP levels in fibroblasts were treated with blank media (NT), 10 ng/mL TGF‐β1 (T), 10 ng/mL TGF‐β1 plus 0.03 μM PDE4 inhibitor roflumilast N‐oxide (T + 0.03R), 0.3 μM roflumilast N‐oxide (T + 0.3R) or 3 μM roflumilast N‐oxide (T + 3R). Data points were plotted as mean ± SEM, *N* = 3, *n* = 9. *P < 0.05 significant increase in cGMP or cAMP detected vs TGF‐β1‐treated TA‐derived fibroblasts, using Student's *t*‐test.

The most potent PDE1 inhibitor (ITI‐214) and PDE4 inhibitor (roflumilast N‐oxide) were selected for cGMP/cAMP analysis. Rhabdomyosarcoma cells treated with 10 μM spermine NONOate were used as a positive control and showed a significant (*P < 0.05) increase in cGMP levels (Fig. 3A). At none of the concentrations tested did spermine NONOate, ITI‐214 nor roflumilast N‐oxide show a significant increase in intracellular cGMP in TA‐derived fibroblasts (Fig. 3). The positive control forskolin (an adenyl cyclase activator), ITI‐214 and roflumilast N‐oxide all significantly (*P < 0.05) increased cAMP levels in concentration‐dependent manners (Fig. 3).

## Discussion

This study is the first to (i) demonstrate the expression of PDE1, PDE7 and PDE8 in TA‐derived fibroblasts, (ii) show that selective PDE4 inhibition can prevent TGF‐β1‐induced myofibroblast transformation, (iii) establish the antifibrotic effect of PDE7 and PDE8 inhibitors and (iv) show that selective PDE1 and PDE4 inhibitors can raise intracellular cAMP in TA‐derived fibroblasts and prevent myofibroblast transformation.

The RT‐qPCR and in‐cell ELISA experiments indicated the expression of PDE1A, PDE1C, PDE4, PDE5A, PDE7B and PDE8B in TA‐derived fibroblasts. PDE4 and PDE5 have previously been shown to be expressed in human TA‐derived fibroblasts [12, 13] and PDE1, PDE7 and PDE8 gene expression has been confirmed in human corpus cavernosum tissue [19].

All the PDE inhibitors tested inhibited myofibroblast transformation in a concentration‐dependent manner. PDE5 inhibitors have previously been shown to prevent myofibroblast transformation in fibroblasts derived from TA [12, 13] and other tissues [20]. Of the other PDE inhibitors tested, the only one that has been previously demonstrated to inhibit myofibroblast transformation in TA‐derived fibroblasts was the pan‐PDE inhibitor pentoxifylline [12].

The PDE1 inhibitor vinpocetine has been shown to suppress TGF‐β1‐induced α‐SMA expression in mouse cardiac fibroblasts [21], whereas there is no published literature investigating the antifibrotic properties of DSR‐141562 and ITI‐214. To date, no selective PDE1 inhibitor has been demonstrated to inhibit myofibroblast transformation in TA‐derived fibroblasts.

As for PDE4 inhibitors, apremilast has been shown to inhibit TGF‐β1‐induced profibrotic gene expression in human skin fibroblasts [22, 23]. Furthermore, roflumilast, roflumilast N‐oxide and rolipram have all been shown to elicit an antifibrotic effect in TGF‐β1‐treated human foetal lung fibroblasts [24, 25]. The current study, however, is the first to demonstrate that selective PDE4 inhibition can prevent TGF‐β1‐induced myofibroblast transformation in TA‐derived fibroblasts.

The literature on the use of PDE7 and PDE8 inhibitors for the treatment of fibrotic diseases is limited, with no published work focusing on PD.

With cGMP/cAMP as the main downstream effectors of PDEs, assays measuring cGMP/cAMP were utilised to confirm the mechanism of action of PDE enzymes. The cGMP assay results showed no significant increase in intracellular cGMP in TA‐derived fibroblasts. This was expected with the PDE4 inhibitor roflumilast N‐oxide since PDE4 is selectively a cAMP‐hydrolysing enzyme [26], whereas PDE1 is a dual substrate PDE (i.e., it metabolises both cAMP and cGMP) [26] therefore the lack of cGMP production by PDE1 inhibitors was surprising. Spermine NONOate, a nitric oxide donor which stimulates the production of cGMP by soluble guanylyl cyclase [26], also did not elicit an increase in cGMP. Rhabdomyosarcoma cells are known to produce cGMP in response to nitric oxide donors [27]; therefore, increased cGMP production by these cells in response to spermine NONOate confirms assay functionality. These results suggest that TA‐derived fibroblasts in our experimental conditions may not have the functional signalling mechanisms necessary for cGMP production.

In contrast, the adenyl cyclase activator forskolin, and both ITI‐214 and roflumilast N‐oxide increased intracellular cAMP in concentration‐dependent manners. Previous studies demonstrated that PDE1/PDE4 inhibitors could prevent myofibroblast transformation by increasing cAMP levels in heart, lung and liver tissues [6, 28, 29]. Currently, only one study [12] has measured the effect of PDE inhibitors on cAMP/cGMP levels in TA‐derived fibroblasts. The authors found that the PDE5 inhibitor sildenafil and the pan‐PDE inhibitor pentoxifylline could respectively raise cGMP and cAMP levels, but at higher concentrations (>100 μM) than those tested in our study.

In this study, we isolated fibroblasts from the non‐plaque tissue using the Nesbit procedure. This was intentional as plaque tissue tends to have a lower cell yield than non‐plaque side (unpublished observations). It would be interesting to compare cells from plaque vs non‐plaque side in future research.

We have previously shown that PDE5 inhibitors prevent transformation of TA‐derived fibroblasts to myofibroblasts [13]. We have also shown a synergy between PDE5 inhibitors and selective oestrogen receptor modulators (SERMs) such as tamoxifen and raloxifene [13]. This synergistic action has led to clinical studies in which a combination of a PDE5 inhibitor and tamoxifen has been shown to halt the progression of fibrosis/plaque formation in patients with acute PD [3, 15, 16]. We have recently observed that PDE5 inhibitors inhibit myofibroblast transformation through the cAMP/protein kinase A pathway, potentially via an off‐target effect, rather than their well‐established PDE5/cGMP/protein kinase G pathway [30]. Further studies will be required to understand the molecular mechanisms involved in the antifibrotic effect of PDE5 inhibitors in acute PD.

In conclusion, this study demonstrated the mRNA and protein expression of PDE1, 4, 5, 7, and 8 isoforms in TA‐derived fibroblasts. Selective inhibitors of these enzymes prevented TGF‐β1‐induced myofibroblast transformation in concentration‐dependent manners, with the PDE1 inhibitor ITI‐214 and PDE4 inhibitors roflumilast and roflumilast N‐oxide showing the greatest potencies. Functional assays confirmed that ITI‐214 and roflumilast N‐oxide both increased intracellular cAMP, but not cGMP, at physiologically relevant concentrations. These results suggest that selective PDE1 and 4 inhibitors can prevent myofibroblast transformation by activation of the cAMP signalling pathway, and thus could be effective in the treatment of acute PD.

### Disclosure of Interest Statement

No conflicts exist for any of the authors.

## Supporting information


**Table S1.** Forward and reverse primers used in RT‐qPCR.
**Table S2.** Primary and secondary antibodies used in ICE assay.
**Figure S1.** Likelihood of gene expression of PDEs decision process.
