# Supplementary material for: Inhibition of phosphodiesterases 1 and 4 prevents myofibroblast transformation in Peyronie's disease
Source: BJU Int. 2024 Dec 23;135(5):810–7. doi: 10.1111/bju.16631 (PMC11975173; doi:10.1111/bju.16631)
Supplement: Supplementary file 1 — Table S1. Forward and reverse primers used in RT‐qPCR. Table S2. Primary and secondary antibodies used in ICE assay. Figure S1. Likelihood of gene expression of PDEs decision process. [file BJU-135-810-s001.docx]

**Supplementary Tables and Figure (Harding et al)**

**Supplementary Table 1: Forward and reverse primers used in RT-qPCR.**

| **Gene** | **Forward Primer** | **Reverse Primer** |
| --- | --- | --- |
| GAPDH | AGCCACATCGCTCAGACA | TGACCAGGCGCCCAATAC |
| PDE1A | CCTCAAAAGCCGAAACTTC | GCATCAGCAATGTGTAACC |
| PDE1B | GTGGATTAAGCTTCGGTCTC | TCAGCTCCTCAATGTTTATCTC |
| PDE1C | GGAAATATAAGAAAACGTCCCAGAG | CTTAAGATCTACCACTGAAGCT |
| PDE2A | CTCTGTGTCCCTGTCATCA | TCGTCGGTGAACAAGTCT |
| PDE3A | GCCAAGAGGATCACAGTC | CAGGAGGTCGGTAATGAG |
| PDE3B | TTGTATTTCCAGAGAACAGATGAT | GCCGAGATCATGCCACTG |
| PDE4A | GCCTCGCACAAGTTCAAA | ACTCTGAGACCTGGTTTCC |
| PDE4B | GGTGATGGCTGATGATAATGT | GTGTTACTGGAAGAACTGTAGG |
| PDE4C | GCCTCCAACAAGTTCAAGC | GAGATGTACTCGGACACCTG |
| PDE4D | CGATCCGACAGCGATTATG | TCAAGTCATCTCCGTGTATATCA |
| PDE5A | ACCAGTGCCTGATGATTCTTA | CGTGGTCTTATATTCTTCAATGG |
| PDE6A | TTTGGACATGGGCATCGT | GTCACAGAAATGCTCATCCTC |
| PDE6B | CAGCGAAGACGAAGATGTGTT | CGCAGTTGTGGAGGTAGC |
| PDE6C | GATGGCAGGGAAGTCAACTTT | CAGGAGGCGTCGGAATCA |
| PDE7A | TGGCAACTTTATACAAGAATACCT | ATGGCAGATGTGAGAATAAGC |
| PDE7B | TTATGCTTCAGATCGCCTTG | ACTCCACTGCTTGCTCAT |
| PDE8A | ATCAGGATAGGCAAGGAGTG | CCAATGACAGGTATTATCTTCACA |
| PDE8B | GGCTCCCATCACAAAGGTTAT | CCAAGGCTTCCGCTACTG |
| PDE9A | AGAGCAGTTCTCAAGAGCAT | TTCTCTAGGACAGCCAAGTG |
| PDE10A | CACCACCGTCTCTGCTTA | GAAATCGTTCATCTCCAAGGAT |
| PDE11A | AGCTCAAACTCACTCCTTCAG | AGATGCTATCAATCCACTCCAG |

**Supplementary Table 2: Primary and secondary antibodies used in ICE assay.**

| **Antibody** | | **Dilution** |
| --- | --- | --- |
| **Primary antibody** | Anti-α-SMA - (Sigma-Aldrich, UK) | 1:3,000 |
|  | Anti-PDE1A - (Abcam, UK) | 1:100 |
|  | Anti-PDE1C - (Novus Biologicals, UK) | 1:100 |
|  | Anti-PDE3A - (Abcam, UK) | 1:100 |
|  | Anti-PDE3B - (Abcam, UK) | 1:100 |
|  | Anti-PDE4 - (Abcam, UK) | 1:100 |
|  | Anti-PDE5A - (Abcam, UK) | 1:500 |
|  | Anti-PDE7A - (Abcam, UK) | 1:100 |
|  | Anti-PDE7B - (Abcam, UK) | 1:250 |
|  | Anti-PDE8A - (Fabgennix, UK) | 1:100 |
|  | Anti-PDE8B - (Fabgennix, UK) | 1:100 |
| **Secondary antibody** | Donkey Anti-Mouse IgG H&L -  (Alexa Fluor® 488) (Abcam, UK) | 1:1,000 |
|  | Donkey Anti-Rabbit IgG (LI-COR, UK) | 1:1,000 |

Melt curves are of good quality

Mean Cq of untreated or TGF-β1-treated cells is below 30

Mean Cq of untreated cells or TGF-β1-treated cells is above 33

Yes

Yes

Yes

No

No

No

Unlikely to be expressed

Likely to be expressed

Unlikely to be expressed

Possibly expressed

**Supplementary Figure 1: Likelihood of gene expression of PDEs decision process.**

Firstly, the melt curves of each gene were assessed, melt curves were determined to be of “good” quality if a single, strong peak was present. A “poor” melt curve was indicative of gene that was unlikely to be expressed. For genes with a “good” melt curve the mean Cqs for untreated and TGF-β1-treated cells were assessed; a Cq of below 30 for either condition was indicative of gene that was likely to be expressed. Genes with Cqs above 30, but below 33 were determined to be possibly expressed. Genes with Cqs above 33 were deemed unlikely to be expressed at biologically relevant levels.
